# Canine tick-borne diseases in pet dogs from Romania

**DOI:** 10.1186/s13071-017-2092-x

**Published:** 2017-03-23

**Authors:** Martin O. Andersson, Conny Tolf, Paula Tamba, Mircea Stefanache, Jonas Waldenström, Gerhard Dobler, Lidia Chițimia-Dobler

**Affiliations:** 1 Center for Ecology and Evolution in Microbial Model Systems (EEMiS), Linnaeus University, SE-391 82 Kalmar, Sweden; 2 Institute for Diagnosis and Animal Health, Bucharest, Romania; 3 PAUMI-VET Private Veterinary Clinics, Snagov, Ilfov County Romania; 4 Bundeswehr Institute of Microbiology, German Center of Infection Research (DZIF) Partner, Neuherbergstrasse 11, D-80937 Munich, Germany

**Keywords:** Dogs, *Canis familiaris*, Vector-borne diseases, Tick-borne diseases, Romania

## Abstract

**Background:**

Tick-borne diseases are of substantial concern worldwide for animals as well as humans. Dogs have been a human companion for millennia, and their significant impact on human life renders disease in dogs to be of great concern. Tick-borne diseases in dogs represent a substantial diagnostic challenge for veterinarians in that clinical signs are often diffuse and overlapping. In addition, co-infections with two or more pathogens enhance this problem further. Molecular methods are useful to disentangle co-infections and to accurately describe prevalence and geographical distribution of tick-borne diseases. At this point, this information is lacking in many areas worldwide. Romania is one such area, where prevalence and distribution of several important pathogens need to be further investigated. To address this, we screened blood samples from 96 sick dogs with molecular methods for eight different pathogens including *Babesia* spp., *Theileria* spp., *Hepatozoon* spp., *Anaplasma* spp., *Ehrlichia* spp., “*Candidatus* Neoehrlichia mikurensis”, *Mycoplasma* spp., and *Borrelia* spp.

**Results:**

As many as 45% (43/96) of the dogs in the study were infected with protozoan parasites. *Babesia canis* was the most frequent of these (28 infected dogs), whereas *Hepatozoon canis* was detected in 15% (14/96) and *Babesia gibsoni* was found in a single sample. Bacterial infection with *Mycoplasma* spp. occurred in 18% (17/96) of the sampled dogs. Obtained bacterial sequences revealed the occurrence of two species: *Mycoplasma canis* and “*Candidatus* Mycoplasma haematoparvum”. In several cases co-infection with protozoan parasites and *Mycoplasma* sp. were detected. All dogs were negative for *Anaplasma* spp., *Ehrlichia* spp., “*Ca.* Neoehrlichia mikurensis”, and for *Borrelia* spp*.*

**Conclusions:**

The results from the present study reinforce the notion that *Babesia canis* is an important pathogen in the Romanian dog population. However, more surprisingly, another protozoan species, *H. canis*, seems to be infecting dogs to a larger extent than previously recognized in Romania. Well-known tick-borne bacterial disease agents such as *Anaplasma* spp. and *Borrelia* spp. were not detected. In contrast, less well-studied bacteria such as hemotropic *Mycoplasma* spp. were detected frequently. Moreover, co-infection might aggravate disease and complicate diagnosis and should be further studied in dogs.

## Background

Dogs are most likely the oldest domestic animal and have for many millennia been a human companion. Diseases in dogs are of great concern, both directly because of costs for owners and suffering in dogs, and indirectly because of the risk for transmission of pathological agents from dogs to humans. Canine vector-borne diseases (CVBDs) are caused by a wide variety of different bacteria, viruses and eukaryotic parasites that are spread by arthropod blood-sucking vectors, mainly ticks and mosquitoes [1]. Globally, the increasing spread of arthropod vectors and associated CVBDs can be explained by several key components, such as ecological and climatic factors and increased mobility of human and animal populations. Combined, these factors have caused a global increase in the distribution of CVBDs [1, 2]. In many areas ticks are the most important arthropod vectors, transmitting a wider variety of CVBDs than any other group of vectors [3, 4]. Several of these pathogens are of substantial zoonotic concern [1]. The probability of dog to human transmission will possibly rise with an increasing number of pet dogs as well as an expanding geographical distribution of several significant vector species.

CVBDs represent a substantial diagnostic challenge for veterinarians, because clinical signs induced by different vector-borne pathogens might be diffuse and overlapping or because diagnostic characteristics are obscured due to co-infections with two or more of these agents [1]. Diagnostic confirmation of CVBDs should include historical exposure to arthropod vectors, compatible clinical signs and physical examination findings, as well as laboratory confirmation with cytological, serological and molecular test results [5]. During the past decade, molecular techniques (e.g. PCR-based methods) have proven to be useful for diagnostic confirmation of many CVBDs, whereas serology and cytology have been used historically in epidemiological surveys or for diagnostic purposes [5].

Romania has a high biodiversity and 25 ixodid tick-species are present, resulting in the presence of many tick-borne pathogens. Despite this, there is only limited information regarding the prevalence of canine tick-borne infections in dogs. Previous studies have concluded that the seroprevalence to a number of well-known tick-borne pathogens were relatively low, with the exception of *B. canis* to which dogs commonly showed an immune response [6]. In addition, studies using molecular techniques have confirmed infection with *Babesia* spp. in dogs [7–9]. Infection with the protozoan parasite *Hepatozoon canis* is known from a single case [10], as well as in four Romanian dogs imported to Germany [11]. *Hepatozoon canis* is one of the most widespread canine tick-borne infections, infecting dogs in large parts of the world [12]. In North-America an additional species of this parasite, *H. americanum*, also causes disease in dogs [13].

The bacterial family *Anaplasmataceae* contains several species that infect various animal species [14]. In Europe, the main causative agent is *Anaplasma phagocytophilum* [15]. In Romania, this species is occurring in ticks [16]*,* while the reported seroprevalence against this bacterium in the Romanian dog population is 5.5% [6]. Moreover, similar results have been reported from other east-European countries [17, 18]. Other species belonging to *Anaplasmataceae* have also been found in Romanian dogs, such as *A. platys* [10], and *E. canis*, to which 2.1% of dogs were seropositive [6].“*Candidatus* Neoehrlichia mikurensis” is another member of the *Anaplasmataceae*. This tick-borne bacterium [19–21] has been detected in several mammal species, including humans [22–25]. Previous studies concerning this bacterium have shown that it has the ability to infect dogs [26] and that it is present in ticks in Romania [16, 27]. However, the scale to what this pathogen is infecting dogs has rarely been investigated. Infections with other bacterial pathogens such as *Borrelia* spp. seem, based on serological analyses, to be relatively rare (0.5%) in the dog population in Romania [6]. Correspondingly, *Mycoplasma* spp. has only been detected once in a dog in Romania using PCR [7]. The aim of this testing was the screening of pet dogs in the area of Snagov, Southern Romania for the occurrence of selected tick-borne protozoan and bacterial infections. A combination of conventional PCR and real-time PCR assays targeting *Babesia* spp., *Theileria* spp., *Hepatozoon* spp., *Anaplasma* spp., *Ehrlichia* spp., “*Ca.* Neoehrlichia mikurensis”, *Mycoplasma* spp., and *Borrelia* spp. in blood samples was used.

## Methods

Blood samples were collected during 2013 and 2014, in Snagov (Iflov County) located in the southern part of Romania by local veterinarians. The tested samples were taken for routine diagnosis from dogs brought to the local veterinary cabinet for diagnosis and treatment of symptoms supposed to be caused by tick-borne infection. The samples were not collected for study reasons. Therefore no epidemiological data were available, except that all dogs had a tick infestation history and were residential and never left the surroundings of Snagov, Ilfov County. The material used in this study consisted of surplus material from clinical investigations. No formal ethical approval was gained. The owners were asked if they agree if the surplus material can be used for additional diagnostic testing. Criteria for the inclusion of samples were epidemiological aspects (exposure to ticks, such as previous infestation and/or residence or visits to tick-infested areas), clinical manifestation (including but not limited to anorexia, depression, fever, jaundice, lethargy/apathy, paleness of external mucosal membranes, weakness), and with or without findings of biochemical abnormalities in hemoglobin, GOT (glutamic-oxaloacetic transaminase), GOP (glutamic-phosphate transaminase), urea and creatinine (all clinical chemistry parameters tested by Reflovet, Roche, Mannheim, Germany). As the sampling of blood from sick dogs was not done as an epidemiological study, different veterinarians provided the samples only with rudimental data from particular dogs, which unfortunately did not allow epidemiological analysis of data in correlation with the results of the PCR testing. DNA was extracted from EDTA-blood using the MagNa Pure LC Instrument and the MagNa Pure LC DNA Isolation Kit I (Roche Applied Science, Mannheim, Germany), using 200 μl of EDTA blood and DNA elution in a final volume of 100 μl.

### Conventional PCR

Conventional PCR amplification of *Babesia/Theileria/Hepatozoon* was performed with the forward primer 5′-GYY TTG TAA TTG GAA TGA TRG-3′ and reverse primer 5′-TAG TTT ATR GTT ARG ACT ACG-3′ that amplify a 411–499 nt fragment of the 18S rRNA gene of *Babesia* spp., *Theileria* spp. and *Hepatozoon* spp. These primers were modified from primers originally designed to amplify only *Babesia* spp. [28].

PCR targeting the 16S rRNA gene of *Anaplasmataceae* was performed with the primers ehr521 and ehr747 according to Pancholi et al. [29], amplifying a 202 bp fragment of the 16S rRNA gene. These primers have been shown to amplify various *Anaplasmataceae* species, including *A. phagocytophilum* and *Ehrlichia chaffeensis*; they also amplify *Rickettsia rickettsii* and *Bartonella henselae* [30]. *Mycoplasma* spp. were amplified with the primers HBT-F and HBT-R [31] that amplify a 595–618 nt fragment of the 16S rRNA gene in various *Mycoplasma* spp. PCR conditions for all assays were as follows; 94 °C for 5 min followed by 35 cycles consisting of 94 °C for 30 s, 55 °C for 30 s and 72 °C for 30 s, followed by 72 °C for 10 minutes*.* PCR reactions were performed in a total reaction volume of 25 μl, which included approximately 20–40 ng of total genomic DNA and by using the Platinum® *Taq* DNA Polymerase (Invitrogen™, Karlsruhe, Germany). All positive PCR amplicons were precipitated and sequenced using the BigDye V 1.1 Cycle Sequencing Kit (Applied Biosystems) and an ABI 3100 sequencer. The sequences were manually edited in the program Geneious 8.1.9 and aligned with relevant sequences published in the GenBank database.

### Real-time PCR

The occurrence of “*Ca.* N. mikurensis” was investigated with a real-time PCR assay targeting the *groEL-*gene [21]. *Borrelia* spp. was amplified with a real-time PCR assay targeting the 16S rRNA gene as previously described [32]. The occurrence of *A. phagocytophilum* was further investigated with a real-time assay with primers from Courtney et al. [33] targeting the *msp2* gene. All real-time PCR reactions were performed in a Light Cycler 480 (Roche, Switzerland) instrument, using the iQ™ SYBR® Green Supermix (Bio-Rad Laboratories, USA). Thermal cycling conditions included an initial denaturation step at 95 °C for 3 min, followed by 45 cycles of 95 °C for 15 s, 60 °C for 30 s and 72 °C for 30 s for all assays.

## Results

Blood samples from 96 dogs were included in the study. The most prevalent pathogen was *Babesia* spp., infecting 30% (29/96) of the investigated dogs. The dominating species amongst these was *B. canis* (28 of 29 cases). Two different *B. canis* genotypes were detected, differing at two nucleotide positions. Twenty-five out of 28 parasite sequences (KY433316) were identical to a *B. canis* sequence found in dogs in Poland and Estonia (KT844900 and KT008057, respectively), whereas the three remaining sequences from Romanian dogs (KY433317) were identical to sequences found in Romanian ticks (KY433323), and also found in dogs from Poland (KT844897). A single nucleotide sequence of *B. gibsoni* (KY433318) was obtained (i.e. corresponding to a prevalence of 1%). This was identical to a sequence from Slovakia, GenBank accession number KP737862 [34]. Previously published sequences from *B. gibsoni* in Romania [9] partially covered a different fragment of the 18S rRNA gene than that reported in the present study. However, all previously published sequences differed on at least one nucleotide position in the 280 bp fragment that overlapped between sequences. Fifteen percent of the dogs (14/96) were infected with *H. canis*. Three different *H. canis* genotypes were found. The most common of these (KY433319), with 10 obtained sequences, were identical to a genotype previously identified in ticks in Romania (KY433326). Another genotype (KY433320), with three additional sequences, was obtained, which differed from the previous genotype, mentioned above, at a single nucleotide position. This genotype was also identical to a sequence previously found in Romanian ticks (KY433327). A third genotype (KY433321) occurred in a single case, and this was identical to a genotype obtained from a fox in Austria (KM115984).

Bacterial infections in the samples were also detected. *Mycoplasma* spp. occurred in 18% of the dogs (17/96) and sequencing revealed two species; *Mycoplasma haemocanis* in 9% (9/96) and “*Candidatus* Mycoplasma haematoparvum” in 8% (8/96), respectively. *Mycoplasma haemocanis* in the present study (KY433883) was identical to a sequence found in a Portuguese dog (GQ129118). The obtained “*Ca.* M. haematoparvum” sequence (KY433884) was identical to a sequence from a dog in Switzerland (EF416569) and also to a sequence obtained from human blood (KF366443). All dogs were negative for *Anaplasma* spp., *Ehrlichia* spp., “*Ca.* Neoehrlichia mikurensis”, and for *Borrelia* spp*.*

Nine cases of co-infections in individual dogs were detected. The most common was concurrent infection with *H. canis* and *M. haemocanis* (3 cases) and *H. canis* and “*Ca,* M. haematoparvum” (3 cases). Co-infection with *B. canis* and *M. haemocanis* occurred in a single case, as did co-infection with *B. canis* and “*Ca.* M. haematoparvum”. Finally, the single dog with *B. gibsoni* infection also harbored *M. haemocanis.*

## Discussion

Results generated in present study showed that as many as 45% (43/96) of dogs suspected to suffer from tick-transmitted infection were infected with apicomplexan parasites, demonstrating the potential impact of these parasites on animal health. More specifically, the study shows that almost one third of sampled dogs were infected with *B. canis*, a result broadly corresponding to previous findings based on serological screenings of Romanian dogs [11, 35]. Furthermore, studies based on molecular screening methods have reported parasite prevalence values up to 71.4% in symptomatic Romanian dogs [9, 11].

*Babesia gibsoni* is generally less common in dogs than *B. canis*; however, a previous study surveying this species in Romania showed that 28.6% of symptomatic dogs were infected [9]. In the present study, only one dog was found to be infected with *B. gibsoni*. This difference is possibly due to the geographical distribution of the parasite in the country, as the previous study was based on samples taken from dogs in the western and north-western parts of the country while dogs in the present study were located in the south. Interestingly, this species seems to colonize new geographical areas where it has not been found before, possibly due to spatial spread of vectors, or alternatively, because of spread in certain susceptible dog breeds [34]. The protozoan parasite *H. canis* has previously been reported in four dogs originating from Romania but imported to Germany [11], as well as in a dog in Romania [10]. In nearby Hungary, prevalence rates exceeding 30% have been reported in shepherd dogs [36], and in Croatia 12% of sampled dogs were infected by *H. canis* [37]. This parasite seems to be more common in dogs in several European countries than previously recognized. Apart from in dogs, *H. canis* is frequently detected in foxes, both in Romania [38] and in several other European countries [39–42].

*Mycoplasma* sp. have been detected in a single dog living in Romania by Hamel and colleagues [11], who screened 29 local pet dogs. Additionally, the same study detected *Mycoplasma* sp. in 16 out of 109 dogs living in Germany but originating from Romania, as well as in one out of 78 dogs originating from Hungary [11]. Although the geographical origin of infection remains unclear, this study concluded that almost ten percent of sampled dogs carried the parasite. The findings by Hamel and colleagues were designated as *Mycoplasma haemocanis* based on the size of the amplified fragment, but the sequences of these fragments were however not determined. In the present study, we report two *Mycoplasma* species confirmed by sequencing: *M. haemocanis* and “*Ca.* Mycoplasma haemoparvum”*.* Both occurred in relatively high numbers, infecting almost one fifth of the dogs in the study, making *Mycoplasma* spp. the second most common pathogen. Travel history of the dogs in the present study was not reported, making it impossible to establish the actual origin of infection. However, the relatively high prevalence of both *Mycoplasma* species does suggest that these are well-established in Romania. Previous publications on *Mycoplasma* spp. in dogs reveal a wide variety of prevalences ranging from 15.4% in France, 9.5% in Italy, 2.5% in Spain and up to 40% in its neighboring country, Portugal [43, 44]. Also, the variation in the prevalence rates between the two species of *Mycoplasma* seems to be extensive based on previous publications. In France, “*Ca.* Mycoplasma haemoparvum”, or a closely related organism, dominated and were found in 15.4% of the investigated dogs, whereas *M. haemocanis* only occurred in 3.3% [43]. In contrast, in Portugal all infections were caused by *M. haemocanis* [44], whereas in Italy a relatively even distribution between the two species occurred [44]. These variations in the prevalence, especially the large variations between neighboring countries such as Spain and Portugal, show the need for large-scale screening efforts in order to better understand the spatial spread of this pathogen throughout Europe, including eastern European countries such as Romania.

None of the other bacteria tested for in this study, i.e. *Anaplasma* spp., *Ehrlichia* spp., “*Ca.* Neoehrlichia mikurensis”, or *Borrelia* spp*.* were detected in samples from the Romanian dogs. Varying levels of seroprevalence rates to these bacteria have previously been reported; Mircean et al. [6] tested 1,146 serum samples from different regions in Romania using ELISA and found that only 129 dogs (11.3%) were positive for any CVBDs with specific seroprevalence values as follows: for *A. phagocytophilum* 5.5%, *E. canis* 2.1%, and *B. burgdorferi* 0.5%. Co-infection with *E. canis* and *A. phagocytophilum* were detected in 2 dogs (0.2%). Immunological methods have an advantage in their ability to reveal the infection history of a particular pathogen, which enables detection of multiple previous infection episodes. This advantage may in part explain the difference in obtained prevalence values between the present study and those based on results obtained with ELISA.

Co-infection with protozoan parasites and *Mycoplasma* spp. were detected in 9% of the dogs in this study. Infection with more than one pathogen in dogs may possible exacerbate clinical manifestations in the infected animal, making the incubation period, clinical outcome and prognosis more unpredictable for the individual dogs [5]. Synergistic or antagonistic effects between co-infecting pathogens in dogs might either enhance or restrict the possibility of a secondary infection with another pathogen, however, such effects need to be studied either by extensive screening of dogs or by performing infection experiments in a controlled environment.

## Conclusions

In the present study, protozoan parasites were commonly detected tick-borne disease agents, with *B. canis* being the most commonly detected species, reinforcing the notion that this is an important parasite in Romanian dogs. The protozoan parasite *H. canis* seems to be infecting dogs in Romania, and possibly in other European countries, to a larger extent than previously recognized and should therefore be considered as an important parasitic agent. The occurrence of this parasite in dog populations in Europe requires further studies. Well known tick-borne bacterial disease agents such as *Anaplasma* spp. and *Borrelia* spp. could not be detected in this study. In contrast, bacterial infection with *Mycoplasma* spp. occurred in a substantial number of the investigated dogs, indicating a rather high prevalence in the dog population in Romania. Health effects of this pathogen need further attention. Moreover, co-infections with protozoan parasites and *Mycoplasma* bacteria could be detected in several dogs. Co-infection might aggravate disease and complicate diagnosis and should be further studied in dogs.

## Abbreviation

CVBD: Canine vector-borne disease
